# Complete mitochondrial genome and assembled DNA barcoding analysis of *Lutjanus fulgens* (Valenciennes, 1830) and its comparison with other *Lutjanus* species

**DOI:** 10.1002/ece3.6542

**Published:** 2020-07-13

**Authors:** Gyamfua Afriyie, Zhongduo Wang, Zhongdian Dong, Christian Ayisi Larbi, Berchie Asiedu, Yusong Guo

**Affiliations:** ^1^ Key Laboratory of Aquaculture in South China Sea for Aquatic Economic Animal of Guangdong Higher Education Institutes Fisheries College Guangdong Ocean University Zhanjiang China; ^2^ Guangdong Provincial Key Laboratory of Pathogenic Biology and Epidemiology for Aquatic Economic Animals Fisheries College Guangdong Ocean University Zhanjiang China; ^3^ Department of Fisheries and Aquatic Resources Management University for Development Studies Tamale Ghana; ^4^ Department of Fisheries and Aquaculture University of Energy and Natural Resources Sunyani Ghana

**Keywords:** *COI* gene, complete mitogenome, DNA barcode, Ghana, management and conservation, phylogeny

## Abstract

*Lutjanus fulgens* (Valenciennes, 1830) is a teleost species classified under the family Lutjanidae which is a native of the Eastern Atlantic Ocean. Though highly commercialized due to its abundance and good taste, the production output has declined in recent years. This is an indication of the need for effective management and conservation measures. However, accurate species identification will ensure strategic management and conservation measure. DNA‐based species identification has proven its reliability in this regard via precise species identification. Several researchers have confirmed the accuracy of DNAbarcode as a species identification tool as well as species phylogeny analysis based on both the complete mitogenome and *COI* gene. Currently, nine specimens of *L. fulgens* were sampled from Ghana and subjected to DNA‐based analysis, namely, complete mitochondrial DNAand *COI* gene (DNA barcoding) analyses. The mitogenomic result revealed that *L. fulgens* is made up of a 16,500 base pairs (bp) mtDNA which consists of 22 transfer RNAs, 13 protein‐coding genes, and two ribosomal RNAs (GenBank Accession Number: MN398650). Furthermore, a sequence polymorphism analysis of the *COI*gene (MN986442–MN986450) detected two haplotypes. These haplotypes were both collected from the same fish landing site which suggests a possible cryptic linage diversity in the *L. fulgens* population at Vodza. According to the phylogeny examination, a close taxonomic relationship exists between *L. fulgens* and *Lutjanus buccanella* caused by a recent evolution termed as sympatric speciation. This study serves as a novel study for this species, building the foundation for future molecular‐based study for this species and as a DNA barcode reference data.

## Graphical abstract

The complete mitochondrial DNA of *Lutjanus fulgens* consists of 16,500 bp of nucleotide which entails 13 protein‐coding genes, two ribosomal RNAs, and 22 transfer RNAs. The phylogeny study indicates that *L. fulgens* is closest related to *Lutjanus buccenella*.

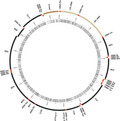

## 1 INTRODUCTION

The family Lutjanidae is highly diverse in the taxonomy and systematics of fisheries. According to FishBase, more than 70 species exist under this family including the Golden African snapper, *Lutjanus fulgens* (Valenciennes, 1830) (https://www.fishbase.se). This species forms a great part of the demersal‐pelagic fauna due to their high abundance in marine environments and commonly found in deeper offshore waters and on rocky bottoms (de Morais et al., 2015; Allen, 1985)*L. fulgens* is a native species in the Eastern Atlantic Ocean (Carpenter, 1992).

Globally, the exponential growth of the human population outweighs the fishery outputs creating a fish deficit (FAO, 2018). Similarly, although highly commercialized species due to its abundance and great taste (de Morais et al., 2015), the production statistics of *L. fulgens* depict a declined production output over the years in Ghana (MoFAD, 2015). This is associated with climate change, poor management measures, and anthropogenic factors such as bad fishing habits, fish habitat degradation, etc. Consequently, this serves as a wake‐up call for fish resource managers to revise and devise management strategies to save this species and the world's fishery at large. The classic approach in managing a fishery resource is dependent on the biological, evolutionary, and ecological knowledge about the particular species to apply a suitable management and conservation strategy. Therefore, accurate species identification and genetic examination are pivotal aspects of the effort of fishery management and conservation.

The complete mitogenome, DNA barcoding, and other genomic‐based information such as microsatellites and random amplified polymorphic DNA are well known genetic markers able to discriminate species and as a source of information for the study of species phylogeny, phylogeography, and evolutionary relationships (Andriyono, Sektiana, Alam, & Kim, 2019; Ceruso, Mascolo, Anastasio, Pepe, & Sordino, 2019; Schmidt, Mcdougall, & Schmidt, 2019). The method of DNA barcoding uses the cytochrome c oxidase I (*COI*) subunit of mitochondrial genes to distinguish species (Bernt et al., 2013). This revolutionary technology which was proposed in 2003 by Hebert and his colleagues, for the identification of species (Hebert, Cywinska, Ball, & DeWaard, 2003) has been used by several researchers and has proven its efficacy as an effective species identification tool (Gan, Grandjean, Jenkins, & Austin, 2019; Iyiola et al., 2018; Ward, Zemlak, Innes, Last, & Hebert, 2005). However, there is little to no genomic information on this species under study. Available works of literature on the genomics of complete mitochondrial DNA (mtDNA) confirm the essential role it plays as a tool for the study of species evolutionary origin as well as evolutionary relationships (Moritz, 1995). This is attributed to its maternal inheritance, different genetic code from other organelles resulting in no intermolecular genetic recombination and high evolutionary rate (Lee, Conroy, Howell, & Kocher, 1995).

Until now, no data have been published on the complete mitochondrial DNA and the DNA barcode of *L. fulgens* though this is not the same story for other Lutjanidaes in other parts of the world. This can be associated with the challenge of the high cost involved in molecular biology studies hence researchers in that region exhibit low interest in this field.

The primary aims of this study were to generate the complete mitochondrial DNA and DNA barcodes of *L. fulgens* and to carry out a molecular‐based study in comparison with other *Lutjanus* species while creating a DNA library for this species via the exploration of DNA barcoding utility as an essential genetic marker for species identification.

## 2 MATERIALS AND METHOD

### 2.1 Specimen sampling and sampling site

A total of 12 specimens of *L. fulgens* were collected from two fish landing sites, *Denu* (6°06′4.54″N, 1°08′51.83″E) and *Vodza* (5°56′20.15″N, 0°59′51.91″E) within the Volta Region of Ghana, West Africa in December 2018. However, nine specimens were successfully sequenced. The samples were identified based on their morphological features (Kwei & Ofori‐Adu, 2005). They were purchased from commercial fishing boats. The muscle specimens were collected and preserved in absolute ethanol and stored in −20°C refrigerator till use. All the specimens are presently stored at the museum of Guangdong Ocean University.

### 2.2 DNA extraction, amplification, and sequencing

#### 2.2.1 Extraction

The traditional method of phenol‐chloroform and proteinase K digestion was utilized to extract the total DNA. DNA quality test using Thermo Scientific NanoDrop 2000 was conducted on the resultant DNA to ascertain the concentration and purity. The integrity of the extracted DNA was detected by agarose gel electrophoresis under UV light.

#### 2.2.2 Amplification

The resultant DNA was subjected to a polymerase chain reaction (PCR) to obtain the *COI* gene. Each of the PCR content was 25 μl and consisted of 8.5 μl of nuclease‐free ddwater, 12.5 μl of 2 × M5 Taq HIFI PCR Mix, 1.5 μl each of primer (FISHCOIF‐5′‐TCAACCAACCACAAAGACATTGGCAC‐3′ and FISHCOIR‐5′‐TAGACTTCTGGGTGGCCAAAGAATCA‐3′) (Ward et al., 2005) and 1 μl of the extracted DNA template. This procedure was repeated for samples that failed to be sequenced at a volume of 50 μl which entailed 18.25 μl of nuclease‐free ddwater, 23.75 μl of 2 × M5 Taq HIFI PCR Mix, 1.5 μl each of primer (FISHCOIF:5 and FISHCOIR) and 5 μl of the DNA template. The thermocycling profile of the PCR comprised the following: 3 min initial denaturing at 94°C, 33 cycles of 30 s at 94°C, annealing for 45 s at 55°C, 1 min extension at 72°C, final extension at 72°C for 5 min and hold at 10°C. The resulting products were observed on 1% agarose gel under UV light.

### 2.3 Sequencing and data analysis

The complete mitogenome and *COI* genes were sequenced by Illumina sequencing and Sanger sequencing, respectively. Following to whole‐genome shotgun strategy, a paired‐end library was built with 400 bp inserts. The paired‐end (2 × 150 bp) sequencing mode under the Illumina Miseq platform was applied to determine the 22,562,826 raw reads (Q20 = 97.63, Q30 = 94.77%). Filtered by AdapterRemoval (version 2) and SOAPec (version 2.01), total 17,906,992 clean reads were used for de novo assembly with A5‐miseq v20150522 and SPAdes v3.9.0 (Coil, Jospin, & Darling, 2015). Lastly, blastn (BLAST v2.2.31+) was used to annotate the contig against the NT database.

The MEGA6.0 software was used to edit the *COI* gene sequences as well as for all multiple sequence alignment analysis. To ensure sequences were devoid of errors and pseudogenes, the length (643 bp) and the quality were assessed. No stop codon was detected, and pseudogenes were deleted. The bioinformatics tools of NCBI Blast and BOLDSystems were utilized to confirm the species morphological identification via cross‐referencing our sequences with the available dataset. Afterward, complete mitogenomes and *COI* genes sequences of other Lutjanidae were downloaded from the GenBank database for the comparison study with our sequences. The DNASP software also was employed to analyze the generated *COI* genes of *L. fulgens* for the presence of nucleotide polymorphism (Rozas, 2009). The intraspecific and interspecific pairwise genetic distance of *COI* genes among species were as well calculated through the Kimura‐2‐parameter (K2P) model (Kimura, 1980; Tamura, Stecher, Peterson, Filipski, & Kumar, 2013). Additionally, the alignment was carried out on the mtDNA and *COI* sequences independently. Subsequently, a phylogenetic examination was done where maximum likelihood (ML) trees were separately constructed using these sequences. Also, a neighbor‐joining (NJ) tree was built for the *COI* sequences of *L. fulgens* generated with *Lutjanus buccanella* (Cuvier, 1828) as an out‐group. This is a member of the family Lutjanidae. A 1,000 bootstrap replication was employed in all the cases.

The complete mitogenome and the *COI* gene sequences generated in the study are deposited at GenBank and can be accessed by MN398650 and MN986442–MN986450, respectively.

## 3 RESULTS

### 3.1 Characteristics of the complete mitochondrial of *L. fulgens*

The complete genome is 16,500 bp in length, with 22 transfer RNA genes (tRNAs), two ribosomal RNA genes, 13 protein‐coding genes, and two noncoding control regions, namely, the origin of light strand replication (OL) and the putative control region (D‐Loop) as in other snappers and distinctive in vertebrates (Andriyono et al., 2019; Kim, Lee, Alam, Lee, & Andriyono, 2019; Taillebois, Crook, Saunders, & Ovenden, 2016). Comparably, this assembled mitogenome was similar to other Lutjanids mitogenome already reported in other studies (Andriyono, Alam, Kwak, & Kim, 2018; Bayona‐Vásquez et al., 2017; Wang, Guo, Wang, Liu, & Liu, 2014; Yamanoue et al., 2007). Furthermore, the number and distribution of these genes are the same as those present in other teleosts such as *Pagellus acarne*, Risso 1827 (Mascolo et al., 2018) and *Dentex dentex*, Linnaeus 1758 (Ceruso et al., 2018) *Lutjanus fulviflamma*, Forsskål, 1775 (Andriyono et al., 2019).

**FIGURE 1:**
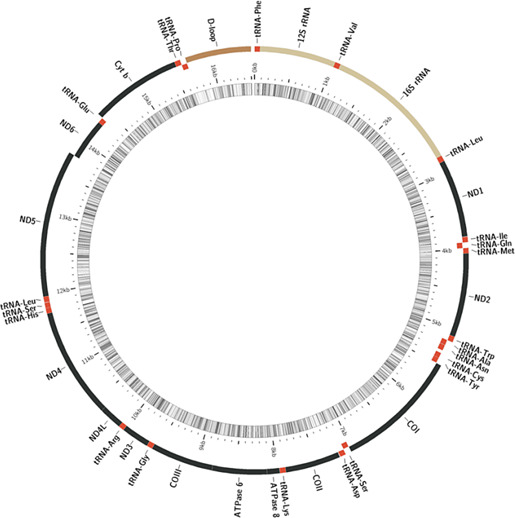
Mitogenomic blueprint of *L. fulgens*

Apart from NADH dehydrogenase subunit 6 (ND6) and eight other tRNA genes (tRNA‐Gln, tRNA‐Ala, tRNA‐Asn, tRNA‐Cys, tRNA‐Tyr, tRNA‐Ser (UGA), tRNA‐Glu and tRNA‐Pro) which were encoded on the light strand, all genes were encoded on the heavy strand (Figure 1).

This is typical in the snappers used in the present study (Kim et al., 2019; Wang et al., 2010) and also other vertebrate**s** (Alam, Petit, Read, & Dove, 2014). The number of nucleotides and composition varied slightly among these Lutjanidaes. The AT‐skew [(A − T)/(A + T)] and GC‐skew[(G − C)/(G + C)] (Table 1) values were close among species.

**TABLE 1:** Summary of the nucleotide composition and skewness of the mitogenome across the *Lutjanus* species under study

Species	Individual base composition				A + T%	AT skew	GC skew
	A %	C %	T %	G %			
*L. fulgens* (Valenciennes, 1830)	27.28	30.75	25.01	16.26	52.29	0.043411742	−0.308232291
*L. russellii* (Bleeker, 1849)	28.16	30.61	25.16	16.07	53.32	0.056264066	−0.311482434
*L. peru* (Nichols & Murphy, 1922)	27.93	30.86	24.85	16.36	52.78	0.058355438	−0.307073274
*L. guttatus* (Steindachner, 1869)	27.82	30.87	24.84	16.46	52.66	0.056589442	−0.30445806
*L. rivulatus* (Cuvier, 1828)	27.88	30.99	24.81	16.32	52.69	0.058265325	−0.310082435
*L. fulviflamma* (Forsskål, 1775)	28.09	30.63	25.13	16.15	53.22	0.055618189	−0.309533989
*L. kasmira* (Forsskål, 1775)	27.86	30.83	25	16.32	52.86	0.054105184	−0.307741251
*L. carponotatus* (Richardson, 1842)	28.02	30.42	25.32	16.22	53.34	0.050618673	−0.304459691
*L. bengalensis* (Bloch, 1790)	28.07	30.23	25.49	16.21	53.56	0.048170276	−0.301894918
*L. argentimaculatus* (Forsskål, 1775)	28.31	31.01	24.73	16.15	53.04	0.067496229	−0.31509754
*L. erythropterus* (Bloch, 1790)	28.14	29.74	25.95	16.17	54.09	0.040488075	−0.295578305
*L. sebae* (Cuvier, 1816)	28.29	30.25	25.43	16.03	53.72	0.053239017	−0.307260156
*L. malabaricus* (Bloch & Schneider, 1801)	27.82	30.84	25.05	16.28	52.87	0.052392661	−0.308998302
*L. vitta* (Quoy & Gaimard, 1824)	28.05	30.27	25.31	16.37	53.36	0.051349325	−0.298027444
*L. johnii* (Bloch 1792)	28.61	30.95	24.79	15.65	53.40	0.071535581	−0.32832618

### 3.2 COI gene identification

A total of nine specimens were successfully sequenced. The bioinformatics search generated different confirmation results from each database, which are NCBI‐BLAST and BOLDSystems. All the successful sequences were positively confirmed as *L. fulgens* according to BOLDSystems. However, the NCBI generated results indicated different species. This is attributed to the absence of data regarding *L. fulgens* on the database, see below (Table 2).

**TABLE 2:** Bioinformatics databases search results on *COI* gene sequences

Specimen Voucher ID	Morphological ID	NCBI ID	Identity (%)	Accession number	BOLD ID	Identity (%)	GenBank Accession number
LFD2	*L. fulgens*	*L. buccanella*	95.64	FJ998465.1	*L. fulgens*	100	MN986442
LFD4	*L. fulgens*	*L. buccanella*	95.67	FJ998465.1	*L. fulgens*	100	MN986443
LFD6	*L. fulgens*	*L. buccanella*	95.36	FJ998465.1	*L. fulgens*	100	MN986444
LFV1	*L. fulgens*	*L. buccanella*	95.77	FJ998465.1	*L. fulgens*	98.91	MN986445
LFV2	*L. fulgens*	*L. buccanella*	95.62	FJ998465.1	*L. fulgens*	99.53	MN986446
LFV3	*L. fulgens*	*L. buccanella*	95.36	FJ998465.2	*L. fulgens*	98.90	MN986447
LFV4	*L. fulgens*	*L. buccanella*	95.24	FJ998465.1	*L. fulgens*	98.91	MN986448
LFV5	*L. fulgens*	*L. buccanella*	95.52	FJ998465.1	*L. fulgens*	99.69	MN986449
LFV6	*L. fulgens*	*L. buccanella*	95.45	FJ998465.1	*L. fulgens*	99.84	MN986450

### 3.3 Pairwise genetic diversity analysis

The intraspecific diversity estimated ranged from 0.000 to 0.014 (see Table 3) while the interspecific ranged from 0.002 to 0.210 (Table 4) where the least value represented the association of *L. purpuerus* and *L. campechanus* whereas *L. vitta* and *L. malabaricus* recorded the highest diversity value. The genetic distance between *L. fulgens* and *L. buccanella*, 0.047, was the third least value in the pairwise analysis after 0.045 matrix between *Lutjanus synagris* and *Lutjanus analis* as the second least.

**TABLE 3:** Intraspecific pairwise genetic distance of *COI* sequence by K2P

Accession number	MN986442	MN986443	MN986444	MN986445	MN986446	MN986447	MN986448	MN986449	MN986450
MN986442									
MN986443	0.003								
MN986444	0.002	0.005							
MN986445	0.012	0.009	0.011						
MN986446	0.002	0.005	0.000	0.011					
MN986447	0.012	0.009	0.014	0.005	0.014				
MN986448	0.002	0.005	0.000	0.011	0.000	0.014			
MN986449	0.000	0.003	0.002	0.012	0.002	0.012	0.002		
MN986450	0.002	0.005	0.002	0.012	0.002	0.014	0.002	0.002	

**TABLE 4:** Interspecies genetic distance based on *COI* gene

S/N	Species	1	2	3	4	5	6	7	8	9	10	11	12	13	14	15	16	17	18	19	20	21	22	23	24
1	*L. buccanella*																								
2	*L. fulgens*	0.047																							
3	*L. russellii*	0.130	0.131																						
4	*L. peru*	0.081	0.079	0.125																					
5	*L. guttatus*	0.060	0.065	0.119	0.067																				
6	*L. rivulatus*	0.119	0.129	0.127	0.121	0.117																			
7	*L. fulviflamma*	0.133	0.135	0.096	0.155	0.131	0.140																		
8	*L. kasmira*	0.123	0.127	0.138	0.125	0.125	0.131	0.146																	
9	*L. carponotatus*	0.130	0.138	0.075	0.141	0.111	0.138	0.075	0.141																
10	*L. bengalensis*	0.133	0.142	0.152	0.144	0.125	0.119	0.142	0.054	0.140															
11	*L. argentimaculatus*	0.142	0.137	0.153	0.111	0.129	0.115	0.146	0.121	0.142	0.140														
12	*L. erythropterus*	0.144	0.168	0.167	0.164	0.155	0.158	0.158	0.154	0.156	0.162	0.156													
13	*L. sebae*	0.184	0.168	0.169	0.159	0.164	0.182	0.166	0.178	0.174	0.184	0.184	0.199												
14	*L. malabaricus*	0.199	0.192	0.174	0.176	0.195	0.171	0.175	0.183	0.178	0.201	0.162	0.183	0.148											
15	*L. vitta*	0.140	0.136	0.108	0.147	0.134	0.137	0.109	0.124	0.102	0.201	0.146	0.174	0.191	0.210										
16	*L. johnii*	0.135	0.148	0.147	0.142	0.148	0.133	0.146	0.151	0.145	0.201	0.140	0.165	0.174	0.197	0.146									
17	*L. alexandrei*	0.122	0.130	0.151	0.134	0.111	0.127	0.119	0.126	0.128	0.201	0.144	0.153	0.174	0.187	0.145	0.140								
18	*L. jocu*	0.117	0.123	0.145	0.126	0.111	0.117	0.121	0.133	0.124	0.201	0.123	0.151	0.172	0.186	0.138	0.133	0.045							
19	*L. cyanopterus*	0.127	0.133	0.144	0.133	0.123	0.130	0.137	0.109	0.135	0.201	0.093	0.151	0.200	0.200	0.133	0.131	0.127	0.121						
20	*L. campechanus*	0.079	0.077	0.131	0.019	0.069	0.123	0.153	0.133	0.145	0.201	0.121	0.166	0.168	0.192	0.147	0.144	0.136	0.128	0.138					
21	*L. vivanus*	0.075	0.069	0.134	0.033	0.071	0.134	0.159	0.136	0.145	0.201	0.123	0.162	0.159	0.190	0.147	0.137	0.136	0.124	0.129	0.038				
22	*L. analis*	0.040	0.060	0.123	0.062	0.040	0.113	0.123	0.119	0.116	0.201	0.119	0.153	0.175	0.190	0.125	0.121	0.120	0.103	0.117	0.067	0.067			
23	*L. purpureus*	0.077	0.075	0.129	0.017	0.071	0.125	0.150	0.131	0.143	0.201	0.119	0.168	0.166	0.190	0.144	0.142	0.134	0.126	0.136	0.002	0.036	0.065		
24	*L. synagris*	0.058	0.067	0.125	0.079	0.019	0.121	0.125	0.129	0.109	0.201	0.146	0.157	0.173	0.190	0.136	0.148	0.119	0.119	0.123	0.081	0.075	0.045	0.083	

Comparatively, the high interspecies values endorse the genetic diversity existing within these Lutjanidaes while the estimated least intraspecific diversity values illustrate the close resemblance as well as confirming the speciation of the specimen from common ancestry.

### 3.4 Multi‐sequence alignment and phylogenetic analysis

**FIGURE 2:**
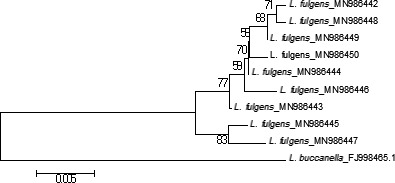
Neighbor‐joining analysis based on *COI* gene sequenced in this study with *L. buccanella a*s an out‐group

**FIGURE 3:**
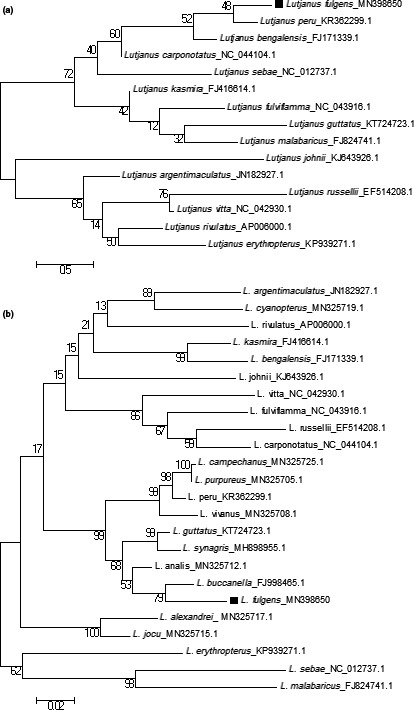
Phylogenetic analysis of (a) complete mtDNA (b) *COI* sequence of *L. fulgens* with other Lutjanidae species by maximum likelihood method

In addition to MEGA multiple sequence alignment, the *COI* genes of *L. fulgens* were again aligned with the GenDoc software. The sequence polymorphism analysis revealed the presence of two haplotypes in the *COI* gene sequences obtained from *Vodza* (MN986445 and MN986447). This suggests the possibility of cryptic linage diversity in the *L. fulgens* population at *Vodza*. Subsequently, these haplotypes formed a monophyletic clade (Figure 2) in the NJ tree while the remaining sequences branched to form different clades. The phylogeny on all the snappers (Figure 3a,b) shows the formation of clades based on sister taxa.

## 4 DISCUSSION

Serving as a good source of cheap animal protein, *L. fulgens* represents a high‐valued economic species in Ghana based on its abundance and good taste (de Morais et al., 2015). According to the MoFAD annual report, the fishery sector provides more than US$1 billion in revenue annually and generates at least 4.5% of the country's Gross domestic product (GDP) (MoFAD, 2015). Nonetheless, the fishery statistics show a continuous reduction in the catches of this species (Asiedu & Nunoo, 2016). This discloses the need for an in‐depth study on the biology, ecology, and evolution of *L. fulgens* for the development of sustainable species‐specific management and conservation strategy to enhance the management of the stock. The precedent action for the materialization of this effort is the accurate identification of this species.

Owing to its efficacy, molecular genetics has earned a considerable world recognition (Hubert et al., 2008; Iyiola et al., 2018), not limited to the field of species identification but also population monitoring, species evolution study as well in the area of forensic science (McKiernan & Danielson, 2017). And as employed in this study, the molecular analysis revealed a similar nucleotide composition, genes, and genes arrangement within the family Lutjanidae (Table 1). The results showing the presence of all the standard genes; 22 tRNA, 13 protein‐coding genes and two rRNA, identified in most teleost and conforms to the standard sets of genes in vertebrates as well (Andriyono et al., 2019; Ceruso et al., 2018; Guo, Bai, Yan, Wang, & Liu, 2014; Kappas, Vittas, Pantzartzi, Drosopoulou, & Scouras, 2016; Shi, Tian, Lin, Huang, & Wang, 2016).

As a species discriminatory marker, the DNA barcodes diversity analysis (Table 3) revealed but a small degree (0.000–0.014) of divergence and the presence of haplotypes within the population of *L. fulgens*. Whereas mtDNA is inherited maternally, haplotypes are alleles passed on from the father to the progeny via Y‐chromosome (Roewer, 2009). Both are used as genetic markers due to their inability to recombine during the crossover. Therefore, the detected haplotypes suggest the preservation of an ancestral allele that did not mutate over the years of evolution. This could aid in Single nucleotide polymorphic (SNP) site identification around the chromosome and it is critical in species discrimination. On the other hand, the interspecific genetic distance analysis (Table 4) produced greater diversity levels as compared with the intraspecific diversity. This confirms a much taxonomic difference among these snappers. Regardless, a marginal genetic diversity between *L. purpureus* and *L campechanus* (0.002) was observed. This recorded as the least interspecific pairwise genetic distance. *L. synagris* and *L. analis* (0.045) and *L. fulgens* and *L. buccanella*, (0.047) also recorded the second and third least matrixes. These species are natives of the western and eastern Atlantic according to FishBase, therefore, indicates sympatric speciation possibly due to genetic drift, polyploidy, hybridization, or mutation from a common ancestor for each paired group (Boddum, 2008).

According to BLAST, the *COI* genes of *L. fulgens* were similar to that of *L. buccanella* between identity percentages of 93.23% to 95.77%. However, due to the lack of complete mtDNA data on *L. buccanella*, *Lutjanus peru* showed a closer relationship with *L. fulgens* based on complete mtDNA ML phylogeny examination (Figure 3a). And the ML analysis based on *COI* genes confirmed that *L. fulgens* shares a much closer relationship with *L. buccanella* as shown in Figure 3b. According to Helfman, Collette, Facey, and Bowen (2009), limited biodiversity exists among the genera of fish inhabiting the western Atlantic, eastern Atlantic, and eastern Pacific. The findings of this research confirmed this conclusion as five species, namely, *L. fulgens*, *L. buccanella*, *L. analis*, *L. synagris*, and *Lutjanus guttatus* formed a paraphyletic clade (Figure 3b). These species occupy the eastern and western Atlantic as well as the eastern Pacific. This suggests cryptic speciation between these species but a more recent evolution between *L. fulgens* and *L. buccanella* (Helfman et al., 2009).

Comparatively, DNA barcoding provides relatively accurate fish identification results with less stress as compared to morphological identification which requires much time and the expertise of skilled taxonomists (Bingpeng et al., 2018; Hubert et al., 2008; Iyiola et al., 2018; Wang et al., 2010). Nonetheless, the expertise of a taxonomist is needed during the sampling of fish for accurate specimen collection. Therefore, the collaboration between both molecular researchers and the traditional morphologic taxonomist is highly encouraged in ichthyofaunal studies as well as other species (Elliott & Davies, 2014) in the effort to generate DNA libraries for all organism by the Consortium for the Barcode of Life (CBOL) (https://www.liquisearch.com) and the International Barcode of Life (iBOL) (https://ibol.org/).

## 5 CONCLUSION

This article focuses on the analysis of the complete mitochondrial genome and *COI* genes of *L. fulgens*. Here, we successfully sequenced the complete mitogenome and nine *COI* genes and subjected them to various bioinformatics analysis. The result regarding the mitogenome depicts the presence of all genes found within other teleost and in a typical vertebrate. Moreover, the DNA polymorphism and multiple sequence alignment revealed the presence of two haplotypes among the *COI* genes. The resultant phylogenetic tree regarding all the Lutjanidaes in the research showed that *L. fulgens* and *L. buccanella* clustered together. Furthermore, all the *COI* genes of *L. fulgens* formed a phylogeny with different clades where the two identified haplotypes formed a monophyletic clade. According to the findings of this study, authors propose further molecular‐based studies on *L. fulgens* and *L. buccanella* together with *L. analis* to fathom the evolutionary relationship between these species.

The findings of this study confirm the accuracy of DNA‐based approach as a tool for species identification. Given that this paper is the first to report on the complete mitogenome and *COI* gene of *L. fulgens*, it lays the foundation for future molecular research on *L. fulgens*. Likewise, it serves as a DNA barcode reference data for correct identification of *L. fulgens* which will assist fishery managers in their quest of duty for effective management strategies decisions.

## CONFLICT OF INTEREST

The authors report no conflicts of interest. The authors alone are responsible for the content and writing of the paper.

## AUTHOR CONTRIBUTIONS

**Gyamfua Afriyie:** Formal analysis (equal); methodology (equal); writing – original draft (lead); writing – review and editing (lead). **Zhongduo Wang:** Conceptualization (equal); formal analysis (equal); project administration (lead). **Zhongdian Dong:** Project administration (equal); visualization (equal). **Christian Ayisi Larbi:** Investigation (equal); resources (equal). **Berchie Asiedu:** Investigation (supporting); resources (supporting). **Yusong Guo:** Conceptualization (equal); funding acquisition (lead); project administration (lead); supervision (lead).

## Supporting information

Table S1‐S2

## Data Availability

The complete mitogenome and the *COI* gene sequences extracted for the study are deposited at GenBank and can be accessed by MN398650 and MN986442–MN986450, respectively. The sampling details of our specimens, as well as the other sequences downloaded from GenBank for the comparison study, are available as supplementary information.
