# Supplementary material for: Complete mitochondrial genome and assembled DNA barcoding analysis of Lutjanus fulgens (Valenciennes, 1830) and its comparison with other Lutjanus species
Source: Ecol Evol. 2020 Jul 13;10(15):7971–80. doi: 10.1002/ece3.6542 (PMC7417232; doi:10.1002/ece3.6542)
Supplement: Supplementary file 1 — Table S1‐S2 [file ECE3-10-7971-s001.docx]

Table S1: Sampling details

| **S/N** | **Specimen Voucher ID** | **Organism** | **Sampling site (GPS Coordinates)** |
| --- | --- | --- | --- |
| 1 | LFD2 | *Lutjanus fulgens* | Denu (6°06' 4.54''N 1°08' 51.83''E) |
| 2 | LFD4 | *Lutjanus fulgens* |  |
| 3 | LFD6 | *Lutjanus fulgens* |  |
| 4 | LFV1 | *Lutjanus fulgens* | Vodza (5°56'20.15''N 0° 59' 51.90''E) |
| 5 | LFV2 | *Lutjanus fulgens* |  |
| 6 | LFV3 | *Lutjanus fulgens* |  |
| 7 | LFV4 | *Lutjanus fulgens* |  |
| 8 | LFV5 | *Lutjanus fulgens* |  |
| 9 | LFV6 | *Lutjanus fulgens* |  |

Table S2: List of snappers selected for the comparison studies

| **Scientific Name** | **English Name** | **Length (bp)** | **GenBank**  **Accession Number** | **Reference** |
| --- | --- | --- | --- | --- |
| *L. russelli*  (Bleeker, 1849) | [Russell's Snapper](http://www.handlinefishing.com/whatsthisfish/snappers/russellssnapper.htm) | 16,505 | EF514208.1 | Wang et al. 2014 |
| *L. peru*  ([Nichols](http://en.wikipedia.org/wiki/John_Treadwell_Nichols) & Murphy[, 1922](http://researcharchive.calacademy.org/research/ichthyology/catalog/getref.asp?id=15356)) | Pacific Red snapper | 16,502 | KR362299.1 | Bayona-Vásquez et al. 2017 |
| [*L. guttatus*](https://www.fishbase.se/Summary/SpeciesSummary.php?ID=1385&genusname=Etelis&speciesname=coruscans)  [(Steindachner](http://en.wikipedia.org/wiki/Franz_Steindachner)[, 1869)](http://researcharchive.calacademy.org/research/ichthyology/catalog/getref.asp?id=20467) | Spotted Rose Snapper | 16,508 | KT724723.1 | Bayona-Vásquez et al. 2017 |
| *L. rivulatus*  [(Cuvier](https://en.wikipedia.org/wiki/Georges_Cuvier)[, 1828)](http://researcharchive.calacademy.org/research/ichthyology/catalog/getref.asp?id=997) | Blubberlip snapper | 16,511 | AP006000.1 | Yamanoue et al. 2007 |
| *L. fulviflamma*  [(Forsskål](http://en.wikipedia.org/wiki/Peter_Forssk%C3%A5l)[, 1775)](http://researcharchive.calacademy.org/research/ichthyology/catalog/getref.asp?id=1351) | Dory snapper | 16,512 | NC_043916.1 | Andriyono et al. 2019 |
| *L. kasmira*  [(Forsskål](http://en.wikipedia.org/wiki/Peter_Forssk%C3%A5l)[, 1775)](http://researcharchive.calacademy.org/research/ichthyology/catalog/getref.asp?id=1351) | Bluestripe snapper | 16,514 | FJ416614.1 | Wang et al. 2010 |
| *L. carponotatus*  [(Richardson](http://en.wikipedia.org/wiki/John_Richardson_(naturalist))[, 1842)](http://researcharchive.calacademy.org/research/ichthyology/catalog/getref.asp?id=21245) | Spanish flag snapper | 16,514 | NC_044104.1 | Kim et al. 2019 |
| [*L. bengalensis*](https://www.fishbase.se/Summary/SpeciesSummary.php?ID=1070&genusname=Lutjanus&speciesname=fulgens)  [(Bloch](http://en.wikipedia.org/wiki/Marcus_Elieser_Bloch)[, 1790)](http://researcharchive.calacademy.org/research/ichthyology/catalog/getref.asp?id=469) | Bengal snapper | 16,511 | FJ171339.1 | Wang et al. 2010 |
| *L. argentimaculatus*  [(Forsskål](http://en.wikipedia.org/wiki/Peter_Forssk%C3%A5l)[, 1775)](http://researcharchive.calacademy.org/research/ichthyology/catalog/getref.asp?id=1351) | Mangrove red snapper | 16,543 | JN182927.1 | Liao et al. 2013 |
| *L. erythropterus* ([Bloch](http://en.wikipedia.org/wiki/Marcus_Elieser_Bloch)[, 1790](http://researcharchive.calacademy.org/research/ichthyology/catalog/getref.asp?id=469)) | Crimson snapper | 16,509 | KP939271.1 | Zhou and Xie 2017 |
| *L. sebae*  [(Cuvier](http://en.wikipedia.org/wiki/Georges_Cuvier)[, 1816)](http://researcharchive.calacademy.org/research/ichthyology/catalog/getref.asp?id=993) | Emperor red snapper | 16,512 | NC_012737.1 | Wang et al. 2010 |
| *L. malabaricus*  [(Bloch](http://en.wikipedia.org/wiki/Marcus_Elieser_Bloch) & [Schneider](http://en.wikipedia.org/wiki/Johann_Gottlob_Theaenus_Schneider)[, 1801)](http://researcharchive.calacademy.org/research/ichthyology/catalog/getref.asp?id=471) | Malabar blood snapper | 16,526 | FJ824741.1 | Wang et al. 2010 |
| *L. vitta*  [(Quoy](http://en.wikipedia.org/wiki/Jean_Rene_Constant_Quoy) & [Gaimard](http://en.wikipedia.org/wiki/Joseph_Paul_Gaimard)[, 1824)](http://researcharchive.calacademy.org/research/ichthyology/catalog/getref.asp?id=3574) | Brownstripe red snapper | 16,498 | NC_042930.1 | Andriyono et al. 2018 |
| *L. johnii*  (Bloch, 1792) | John’s snapper | 16,596 | KJ643926.1 | Taillebois et al., 2016 |
